# Multiview Discriminative Geometry Preserving Projection for Image Classification

**DOI:** 10.1155/2014/924090

**Published:** 2014-03-09

**Authors:** Ziqiang Wang, Xia Sun, Lijun Sun, Yuchun Huang

**Affiliations:** School of Information Science and Engineering, Henan University of Technology, Zhengzhou 450001, China

## Abstract

In many image classification applications, it is common to extract multiple visual features from different views to describe an image. Since different visual features have their own specific statistical properties and discriminative powers for image classification, the conventional solution for multiple view data is to concatenate these feature vectors as a new feature vector. However, this simple concatenation strategy not only ignores the complementary nature of different views, but also ends up with “curse of dimensionality.” To address this problem, we propose a novel multiview subspace learning algorithm in this paper, named multiview discriminative geometry preserving projection (MDGPP) for feature extraction and classification. MDGPP can not only preserve the intraclass geometry and interclass discrimination information under a single view, but also explore the complementary property of different views to obtain a low-dimensional optimal consensus embedding by using an alternating-optimization-based iterative algorithm. Experimental results on face recognition and facial expression recognition demonstrate the effectiveness of the proposed algorithm.

## 1. Introduction

Many computer vision and pattern recognition applications involve processing data in a high-dimensional space. Directly operating on such high-dimensional data is difficult due to the so-called “curse of dimensionality.” For computational time, storage, and classification performance considerations, dimensionality reduction (DR) techniques provide a means to solve this problem by generating a succinct and representative low-dimensional subspace of the original high-dimensional data space. Over the past two decades, many dimensionality reduction algorithms have been proposed and successfully applied to face recognition [1]. The most representative ones are principal component analysis (PCA) and linear discriminant analysis (LDA) [2].

PCA is an unsupervised dimensionality reduction method, which aims to project the high-dimensional data into a low-dimensional subspace spanned by the leading eigenvectors of a covariance matrix. LDA is supervised and its goal is to pursue a low-dimensional subspace by maximizing the ratio of between-class variance to within-class variance. Due to the utilization of label information, LDA usually outperforms PCA for classification tasks when sufficient labeled training data are available. While these two algorithms have attained reasonable good performance in pattern classification, they may fail to discover a highly nonlinear submanifold embedded in the high-dimensional ambient space as they seek only a compact Euclidean subspace for data representation and classification [3].

Recently, there has been considerable interest in manifold learning algorithms for dimensionality reduction and feature extraction. The basic consideration of these algorithms is that the high-dimensional data may lie on an intrinsic nonlinear low-dimensional manifold. In order to detect the underlying manifold structure, nonlinear dimensionality reduction algorithms such as ISOMAP [4], locally linear embedding (LLE) [5], and Laplacian eigenmap (LE) [6] have been proposed. All of these algorithms are defined only on the training data, and the issue of how to map new testing data remains difficult. Therefore, they cannot be applied to classification problem directly. To overcome the above so-called out-of-sample problem, He and Niyogi [7] developed the locality preserving projection (LPP), in which the linear projection function is adopted for mapping new data samples. As LPP is originally unsupervised, some recent attempts have exploited the discriminant information and derived many discriminant manifold learning algorithms to enhance the classification performance. The representative algorithms include local discriminant embedding (LDE) [8], locality sensitive discriminant analysis (LSDA) [9], margin Fisher analysis (MFA) [10], local Fisher discriminant analysis (LFDA) [11], and discriminative geometry preserving projection (DGPP) [12]. Despite having different assumptions, all these algorithms can be unified into a general graph embedding framework (GEF) [10] with different constraints. While these algorithms have utilized both local geometry and the discriminative information for dimensionality reduction and achieved reasonably good performance in different pattern classification tasks, they assume that the data are represented in a single vector. They can be regarded as single-view-based methods and thus cannot handle data described by multiview features directly. In many practical pattern classification applications, different views (visual features) have their own specific statistical properties, and each view represents the data partially. To address this problem, the traditional solution for multiple view data is to simply concatenate vectors of different views into a new long vector and then apply dimensionality reduction algorithms directly on the concatenated vector. However, this concatenation ignores the diversity of multiple views and thus cannot explore the complementary nature and specific statistical properties of different views. Recent studies have provided convincing evidence of this fact [13–15]. Hence, it is more reasonable to assign different weights to different views (features) for feature extraction and classification. In computer vision and machine learning research, many works have shown that leveraging the complementary nature of the multiple views can better represent the data for feature extraction and classification [13–15]. Therefore, an efficient manifold learning algorithm that can cope with multiview data and place proper weights on different views is of great interest and significance.

Motivated by the above observations and reasons, we propose unifying different views under a discriminant manifold learning framework called multiview discriminative geometry preserving projection (MDGPP). Under each view, we can implement the discrimination and local geometry preservations as those used in discriminative geometry preserving projection (DGPP) [12]. Unifying different views in such a multiview discriminant manifold learning framework is meaningful, since data with different features can be appropriately integrated to further improve the classification performance. Specifically, we first implement the discrimination preservation by maximizing the average weighted pairwise distance between samples in different classes and simultaneously minimizing the average weighted pairwise distance between samples in the same class. Meanwhile, the local geometry preservation is implemented by minimizing the reconstruction error of samples in the same class. Then, we learn a low-dimensional feature subspace by utilizing both intraclass geometry and interclass discrimination information, such that the complementary nature of different views (features) can be fully exploited when classification is performed in the derived feature subspace. Experimental results on face recognition and facial expression recognition are presented to demonstrate the effectiveness of the proposed algorithm.

The remainder of the paper is organized as follows.  Section 2 reviews the related works.  Section 3 presents the details of the proposed MDGPP algorithm. Experimental results on face recognition are presented in Section 4, and the concluding remarks are provided in Section 5.

## 2. Related Works

Multiview learning is one important topic in the machine learning and pattern recognition communities. In such a setting, view weight information is introduced to measure the importance of different features in characterizing data, and different weights reflect different contribution to the learning process. The aim of multiview learning is to exploit more complementary information of different views rather than only a single view to further improve the learning performance. The traditional solution for multiview data is to concatenate all features into one vector and then conduct machine learning for such feature space. However, this solution is not optimal as these features usually have different physical properties. Simply concatenating them will ignore the complementary nature and specific statistical properties of different views, and thus causing performance degradation. In addition, this simple concatenation will end up with the curse of dimensionality problem for the subsequent learning task.

In order to perform multiview learning, much effort has been focused on multiview metric learning [14], multiview classification and retrieval [16], multiview clustering [15], and multiview semisupervised learning [17]. All these approaches demonstrated that the learning performance can be significantly enhanced if the complementary nature of different views is exploited and all views are appropriately integrated. It is very natural that multiview learning idea should also be considered in dimensionality reduction. However, most of the existing dimensionality reduction algorithms are designed only for single view data and cannot cope with multiview data directly. To address this problem, Long et al. [18] first proposed multiple view spectral embedding (MVSE) method. MVSE performs a dimensionality reduction process on each view independently, and then based on the obtained low-dimensionality representation, it constructs a common low-dimensional embedding that is close to each representation as much as possible. Although MVSE allow selecting different dimensionality reduction algorithms for each view, the original multiview data are invisible to the final learning process. Thus, MVSE cannot well explore the complementary information of different views. Xia et al. [19] proposed multiview spectral embedding (MSE) method to find low-dimensional and sufficiently smooth embedding based on the patch alignment framework [20]. However, MSE ignores the flexibility of allowing shared information between subset of different views owing to the global coordinate alignment process. To unify different views for dimensionality reduction under a probabilistics, Xie et al. [21] extended the stochastic neighbor embedding (SNE) to its multiview version and proposed multiview stochastic neighbor embedding (MSNE). Although MSNE operates on a probabilistic framework, it is an unsupervised method and its classification abilities may be limited since the class label information is not used in the learning process. More recently, inspired by the recent advances of sparse coding technique, Han et al. [22] proposed spectral sparse multiview embedding (SSMVE) method to deal with dimensionality reduction for multiview data. Although SSMVE can impose sparsity constraint on the loading matrix of multiview dimensionality reduction, it is unsupervised and does not explicitly consider the manifold structure on which the high dimensional data possibly reside. In the next section, focusing on the manifold learning and pattern classification, we propose a novel multiview discriminative geometry preserving projection (MDGPP) for multiview dimensionality reduction, which explicitly considers the local manifold structure and discriminative information as well as the complementary characteristics of different views in high-dimensional data.

## 3. Multiview Discriminative Geometry Preserving Projection (MDGPP)

In this section, we propose a new manifold learning algorithm called multiview discriminative geometry preserving projection (MDGPP), which aims to find a unified low-dimensional and sufficiently smooth embedding over all views simultaneously. To better explain the algorithm details of the proposed MDGPP, we introduce some important notations used in the remainder of this paper. Capital letters such as *X* denote data matrices, and *X*_*ij*_ represents the (*i*, *j*) entry of *X*. Lower case letters such as *x* denote data vectors, and *x*_*i*_ represents the *i*th data element of *x*. Superscript (*i*) such as *X* ^(*i*)^ and *x* ^(*i*)^ represents data from the *i*th view, respectively. Based on these notations, MDGPP can be described as follows according to the DGPP framework [12].

Given a multiview data set with *n* data samples and each with *m* feature representations, that is, *X* = {*X* ^(*i*)^=[*x*_1_^(*i*)^,*x*_2_^(*i*)^,…,*x*_*n*_^(*i*)^]∈*R* ^*p*_*i*_×*n*^}_*i*=1_^*m*^, wherein *X* ^(*i*)^ represents the feature matrix for the *i*th view, the aim of MDGPP is to find a projective matrix *W* ∈ *R* ^*p*_*i*_×*d*^ to map *X* ^(*i*)^ into a low-dimensional representation *Y* ^(*i*)^ through *Y* ^(*i*)^ = *W* ^*T*^ *X* ^(*i*)^, where *d* denotes the dimension of low-dimensional feature representation and satisfies *d* < *p*_*i*_  (1 ≤ *i* ≤ *m*). The workflow of MDGPP can be simply described as follows. First, MDGPP builds a part optimization for a sample on a single view by preserving both the intraclass geometry and interclass discrimination. Afterward, all parts of optimization from different views are unified as a whole via view weight coefficients. Then an alternating-optimization-based iterative algorithm is derived to obtain the optimal low-dimensional embedding from multiple views.

Given the *i*th view *X* ^(*i*)^ = [*x*_1_^(*i*)^, *x*_2_^(*i*)^,…, *x*_*n*_^(*i*)^] ∈ *R* ^*p*_*i*_×*n*^, MDGPP first makes an attempt to preserve discriminative information in the reduced low-dimensional space by maximizing the average weighted pairwise distance between samples in different classes and simultaneously minimizing the average weighted pairwise distance between samples in the same class on the *i*th view. Thus, we have

(1) argmax⁡yj(i)∑j,t=1nhjt(i)||yj(i)−yt(i)||2 =argmax⁡yj(i)∑j,t=1nhjt(i)||yj(i)−yt(i)||2 =argmax⁡yj(i) Tr⁡(∑j,t=1nhjt(i)(yj(i)−yt(i))(yj(i)−yt(i))T) =argmax⁡yj(i) Tr⁡(∑j,t=1nhjt(i)(yj(i)(yj(i))T−yt(i)(yj(i))T           −yj(i)(yt(i))T+yt(i)(yt(i))T)) =arg max 2Tr(Y(i)D(i)(Y(i))T−Y(i)H(i)(Y(i))T) =arg max 2Tr(Y(i)L(i)(Y(i))T) =arg max 2Tr(UTX(i)L(i)(X(i))TU),

where *Tr*⁡( ) denotes the trace operation of matrix, *L* ^(*i*)^( = *D* ^(*i*)^ − *H* ^(*i*)^) is the graph Laplacian on the *i*th view, *D* ^(*i*)^ is a diagonal matrix with its element *D*_*jj*_^(*i*)^ = ∑_*t*_ *H*_*jt*_^(*i*)^ on the *i*th view, and *H* ^(*i*)^ = [*h*_*jt*_^(*i*)^]_*j*,*t*=1_^*n*^ is the weighting matrix which encodes both the distance weighting information and the class label information on the *i*th view

(2) $$\begin{array}{c} h_{jt}^{\left(i\right)}=\{\begin{array}{cc} c_{jt}\left(\frac{1}{n}-\frac{1}{n_{l}}\right), & \text{if}\;l_{j}=l_{t}=l\\ \frac{1}{n}, & \text{if}\;l_{j}\neq l_{t}, \end{array} \end{array}$$

where in *l*_*j*_ is the class label of sample *x*_*j*_^(*i*)^, *n*_*l*_ is the number of samples belonging to the *l*th class, and *c*_*jt*_ is set as exp⁡(−||*x*_*j*_^(*i*)^ − *x*_*t*_^(*i*)^||/*δ* ^2^) according to LPP [7] for locality preservation.

Second, we try to implement the local geometry preservation by assuming that each sample *x*_*j*_^(*i*)^ can be linearly reconstructed by the samples *x*_*t*_^(*i*)^ which share the same class label with *x*_*j*_^(*i*)^ on the *i*th view. Thus, we can obtain the reconstruction coefficient *w*_*jt*_^(*i*)^ by minimizing the reconstruction error ∑_*j*=1_^*n*^||*ε*_*j*_||^2^ on the *i*th view; that is,

(3) argmin⁡wjt∑j=1n||εj||2  =argmin⁡wjt∑j=1n||xj(i)−∑t:lt=ljwjt(i)xt(i)||2

under the constraint

(4) $$\begin{array}{c} \sum_{t:l_{t}=l_{j}}w_{jt}^{\left(i\right)}=1,\;\;w_{jt}^{\left(i\right)}=0\;\text{for}\;\;l_{t}\neq l_{j}. \end{array}$$

Then, by solving (3) and (4), we have

(5) $$\begin{array}{c} w_{j}^{\left(i\right)}=\frac{\sum_{p}C_{j,p}^{-1}}{\sum_{p,q}C_{p,q}^{-1}}, \end{array}$$

where *C*_*p*,*q*_ = (*x*_*j*_^(*i*)^−*x*_*p*_^(*i*)^)^*T*^(*x*_*j*_^(*i*)^ − *x*_*q*_^(*i*)^) denotes the local Gram matrix and *l*_*p*_ = *l*_*q*_ = *l*_*j*_.

Once obtaining the reconstruction coefficient *w*_*jt*_^(*i*)^ on the *i*th view, then MDGPP aims to reconstruct *y*_*j*_^(*i*)^( = *U* ^*T*^ *x*_*j*_^(*i*)^) from *y*_*t*_^(*i*)^( = *U* ^*T*^ *x*_*t*_^(*i*)^)  (where  *l*_*t*_ = *l*_*j*_) with *w*_*jt*_^(*i*)^ in the projected low-dimensional space; thus we have

(6) argmin⁡yj(i)∑j=1n||yj(i)−∑t:lt=ljwjt(i)yt(i)||2 =argmin⁡yj(i)∑j=1n||yj(i)−∑t:lt=ljwjt(i)yt(i)||2 =argmin⁡yj(i) Tr⁡(∑j=1n(yj(i)−∑t:lt=ljwjt(i)yt(i))         ×(yj(i)−∑t:lt=ljwjt(i)yt(i))T) =arg min 2Tr(Y(i)I(i)(Y(i))T−Y(i)W(i)(Y(i))T) =arg min 2Tr(Y(i)(I(i)−W(i))(Y(i))T) =arg min 2Tr(UTX(i)(I(i)−W(i))(X(i))TU),

where *I* ^(*i*)^ is an identity matrix defined on the *i*th view, and *W* ^(*i*)^ = [*w*_*jt*_^(*i*)^]_*j*,*t*=1_^*n*^ is the reconstruction coefficient matrix on the *i*th view.

As a result, by combining (1) and (6) together, the part optimization for *X* ^(*i*)^ is

(7) argmax⁡yj(i)(∑j,t=1nhjt(i)||yj(i)−yt(i)||2      −λ∑j=1n||yj(i)−∑t:  lt=ljwjt(i)yt(i)||2) =arg max Tr⁡(UTX(i)L(i)(X(i))TU         −λUTX(i)(I(i)−W(i))(X(i))TU) =arg max Tr⁡(UT(X(i)L(i)(X(i))T            −λX(i)(I(i)−W(i))(X(i))T)U) =arg max Tr⁡(UTQ(i)U),

where *Q* ^(*i*)^ = *X* ^(*i*)^ *L* ^(*i*)^(*X* ^(*i*)^)^*T*^ − *λX* ^(*i*)^(*I* ^(*i*)^ − *W* ^(*i*)^)(*X* ^(*i*)^)^*T*^, and *λ* is a tradeoff coefficient which is empirically set as 1 in this experiment.

Based on the local manifold information encoded in *L* ^(*i*)^ and *W* ^(*i*)^, (7) aims at finding a sufficiently smooth low-dimensional embedding *Y* ^(*i*)^( = *U* ^*T*^ *X* ^(*i*)^) by preserving the interclass discrimination and intraclass geometry on the *i*th view.

Because multiviews could provide complementary information in characterizing data from different viewpoints, different views certainly have different contributions to the low-dimensional feature subspace. In order to well discover the complementary information of data from different views, a nonnegative weighted set *σ* = [*σ*_1_, *σ*_2_,…, *σ*_*m*_] is imposed on each view independently. Generally speaking, the larger *σ*_*i*_ is, the more the contribution of the view *X* ^(*i*)^ is made to obtain the low-dimensional feature subspace. Hence, by summing over all parts of optimization defined in (7), we can formulate MDGPP as the following optimization problem:

(8) $$\begin{array}{c} \arg\underset{U,\sigma }{\max}\overset{m}{\underset{i=1}{\sum }}\sigma_{i}\mathrm{Tr}\left(U^{T}Q^{\left(i\right)}U\right) \end{array}$$

subject to

(9) $$\begin{array}{c} U^{T}U=I,\;\;\overset{m}{\underset{i=1}{\sum }}\sigma_{i}=1,\;\sigma_{i}\geq 0. \end{array}$$

The solution to *σ* in (8) subject to (9) is *σ*_*k*_ = 1 corresponding to the maximum *Tr*⁡(*U* ^*T*^ *Q* ^(*i*)^ *U*) over different views, and *σ*_*k*_ = 0 otherwise, which means that only the best view is finally selected by this method. Consequently, this solution cannot meet the demand for exploring the complementary characteristics of different views to get a better low-dimensional embedding than that based on a single view. In order to avoid this problem, we set *σ*_*i*_ ← *σ*_*i*_^*r*^ with *r* > 1 by following the trick utilized in [16–19]. In this condition, ∑_*i*=1_^*m*^ *σ*_*i*_^*r*^ = 1 achieves its maximum when *σ*_*i*_ = 1/*m* according to ∑_*i*=1_^*m*^ *σ*_*i*_ = 1 and *σ*_*i*_ ≥ 0. Similarly *σ*_*i*_ for different views can be obtained by setting *r* > 1; thus each view makes a specific contribution to obtaining the final low-dimensional embedding. Consequently, the new objective function of MDGPP can be defined as follows:

(10) $$\begin{array}{c} \arg\underset{U,\sigma }{\max}\overset{m}{\underset{i=1}{\sum }}\sigma_{i}^{r}\mathrm{Tr}\left(U^{T}Q^{\left(i\right)}U\right) \end{array}$$

subject to

(11) $$\begin{array}{c} U^{T}U=I,\;\;\overset{m}{\underset{i=1}{\sum }}\sigma_{i}=1,\;\sigma_{i}\geq 0. \end{array}$$

The above optimization problem is a nonlinearly constrained nonconvex optimization problem, so there is no direct approach to find its global optimal solution. In this paper, we derive an alternating-optimization-based iterative algorithm to find a local optimal solution. The alternating optimization iteratively updates the projection matrix *U* and weight vector *σ* = [*σ*_1_, *σ*_2_,…, *σ*_*m*_].

First, we update *σ* by fixing *U*. The optimal problem (10) subject to (11) becomes

(12) $$\begin{array}{c} \arg\underset{U,\sigma }{\max}\overset{m}{\underset{i=1}{\sum }}\sigma_{i}^{r}\mathrm{Tr}\left(U^{T}Q^{\left(i\right)}U\right) \end{array}$$

subject to

(13) $$\begin{array}{c} \overset{m}{\underset{i=1}{\sum }}\sigma_{i}=1,\;\sigma_{i}\geq 0. \end{array}$$

Following the standard Lagrange multiplier, we construct the following Lagrangian function by incorporating the constraint (13) into (12):

(14) $$\begin{array}{c} L\left(\sigma ,\lambda \right)=\overset{m}{\underset{i=1}{\sum }}\sigma_{i}^{r}\mathrm{Tr}\left(U^{T}Q^{\left(i\right)}U\right)-\lambda \left(\overset{m}{\underset{i=1}{\sum }}\sigma_{i}-1\right), \end{array}$$

where the Lagrange multiplier *λ* satisfies *λ* ≥ 0.

Taking the partial derivation of the Lagrangian function *L*(*σ*, *λ*) with respect to *σ*_*i*_ and *λ* and setting them to zeros, we have

(15) $$\begin{array}{c} \frac{\partial L\left(\sigma ,\lambda \right)}{\partial \sigma_{i}}=r\sigma_{i}^{r-1}\mathrm{Tr}\left(U^{T}Q^{\left(i\right)}U\right)-\lambda =0, \end{array}$$

(16) $$\begin{array}{c} \frac{\partial L\left(\sigma ,\lambda \right)}{\partial \lambda }=\overset{m}{\underset{i=1}{\sum }}\sigma_{i}-1=0. \end{array}$$

Hence, according to (13) and (14), the weight coefficient *σ*_*i*_ can be calculated as

(17) $$\begin{array}{c} \sigma_{i}=\frac{{\left(1/\mathrm{Tr}\left(U^{T}Q^{\left(i\right)}U\right)\right)}^{1/\left(r-1\right)}}{\sum_{i=1}^{m}{\left(1/\mathrm{Tr}\left(U^{T}Q^{\left(i\right)}U\right)\right)}^{1/\left(r-1\right)}}. \end{array}$$

Then, we can make the following observations according to (15): If *r* → *∞*; then the values of different *σ*_*i*_ will be close to each other. If *r* → 1, then only *σ*_*i*_ = 1 corresponding to the maximum *Tr*⁡(*U* ^*T*^ *Q* ^(*i*)^ *U*) over different views, and *σ*_*i*_ = 0 otherwise. Thus, the choice of *r* should respect to the complementary property of different views. The effect of the parameter *r* will be discussed in the later experiments.

Second, we update *U* by fixing *σ*. The optimal problem (10) subject to (11) can be equivalently transformed into the following form:

(18) $$\begin{array}{c} \arg\underset{U}{\max}\;\mathrm{Tr}\left(U^{T}QU\right) \end{array}$$

subject to

(19) $$\begin{array}{c} U^{T}U=I, \end{array}$$

where *Q* = ∑_*i*=1_^*m*^ *σ*_*i*_^*r*^ *Q* ^(*i*)^. Since *Q* ^(*i*)^ defined in (7) is a symmetric matrix, *Q* is also a symmetric matrix.

Obviously, the solution of (18) subject to (19) can be obtained by solving the following standard eigendecomposition problem

(20) $$\begin{array}{c} QU=\lambda U. \end{array}$$

Let the eigenvectors *U*_1_, *U*_2_,…, *U*_*d*_ be solutions of (20) ordered according to eigenvalues *λ*_1_ > *λ*_2_ > ⋯>*λ*_*d*_. Then, the optimal projection matrix *U* is given by *U* = [*U*_1_, *U*_2_,…, *U*_*d*_]. Now, we discuss how to determine the reduced feature dimension *d* by using the Ky Fan theorem [23].

*Ky Fan Theorem*. Let *H* be a symmetric matrix with eigenvalues *λ*_1_ ≥ *λ*_2_ ≥ ⋯≥*λ*_*n*_ and the corresponding eigenvectors *U* = [*U*_1_, *U*_2_,…, *U*_*n*_]. Then

(21) $$\begin{array}{c} \lambda_{1}+\lambda_{2}+\cdots +\lambda_{k}=\arg\underset{X^{T}X=I}{\max}\mathrm{Tr}\left(X^{T}HX\right). \end{array}$$

Moreover, the optimal X* is given by X* = *U* = [*U*_1_, *U*_2_,…, *U*_*k*_]*R*, where *R* is an arbitrary orthogonal matrix.

From the above Ky Fan theorem, we can make the following observations. The optimal solution to (18) subject to (19) is composed of the largest *d* eigenvectors of the matrix *Q*, and the optimal value of objective function (18) equals the sum of the largest *d* eigenvalues of the matrix *Q*. Therefore, the optimal reduced feature dimension *d* is equivalent to the number of positive eigenvalues of the matrix *Q*.

Alternately updating *σ* and *U* by solving (17) and (20) until convergence, we can obtain the final optimal projection matrix *U* for multiple views. A simple initialization for *σ* could be *σ* = [1/*m*,…, 1/*m*]. According to the aforementioned statement, the proposed MDGPP algorithm is summarized as follows.

**Algorithm 1 (MDGPP algorithm)**

*Input.* A multiview data set *X* = {*X* ^(*i*)^∈*R* ^*p*_*i*_×*n*^}_*i*=1_^*m*^, the dimension of the reduced low-dimensional subspace *d*  (*d* < *p*_*i*_, 1 ≤ *i* ≤ *m*), tuning parameter *r* > 1, iteration number *T*_max⁡_, and convergence error *ε*.

*Output.* Projection matrix *U*.

*Algorithm.*

**Step 1**

Simultaneously consider both intraclass geometry and interclass discrimination information to calculate *Q* ^(*i*)^ for each view according to (7).

**Step 2 (initialization)**

 (1) Set *σ* = [1/*m*, 1/*m*,…, 1/*m*];
 (2) obtain *U* ^0^ by solving the eigendecomposition problem (20).

**Step 3 (local optimization)**

For *t* = 1,2,…, *T*_max⁡_

 (1) calculate *σ* as shown in (17);
 (2) solve the eigenvalue equation in (20);
 (3) sort their eigenvectors *U*_1_, *U*_2_,…, *U*_*d*_ according to their corresponding eigenvalues: *λ*_1_ > *λ*_2_ > ⋯>*λ*_*d*_, and obtain *U* ^*t*^ = [*U*_1_, *U*_2_,…, *U*_*d*_];
 (4) if *t* > 2 and |*U* ^*t*^ − *U* ^*t*−1^| < *ε*, then go to Step 4.

**Step 4 (output projection matrix)**

Output the final optimal projection matrix *U* = *U* ^*t*^.

We now briefly analyze the computational complexity of the MDGPP algorithm, which is dominated by three parts. One is for constructing the matrix *Q* ^(*i*)^ for different views. As shown in (7), the computational complexity of this part is *O*((∑_*i*=1_^*m*^ *p*_*i*_) × *n* ^2^). In addition, each iteration involves computing view weight *σ* and solving a standard eigendecomposition problem; the computational complexity of running two parts in each iteration is *O*((*m* + *d*) × *n* ^2^) and *O*(*n* ^3^), respectively. Therefore, the total computational complexity of MDGPP is *O*((∑_*i*=1_^*m*^ *p*_*i*_) × *n* ^2^ + ((*m* + *d*) × *n* ^2^ + *n* ^3^) × *T*_max⁡_), where *T*_max⁡_ denotes the iteration number and is always set to less than five in all experiments.

## 4. Experimental Results

In this section, we evaluate the effectiveness of our proposed MDGPP algorithm for two image classification tasks including face recognition and facial expression recognition. Two widely used face databases including AR [24] and CMU PIE [25] are employed for face recognition evaluation, and the well-known Japanese female facial expression (JAFFE) [26] database is used for facial expression recognition evaluation. We also compare the proposed MDGPP algorithm with some traditional single-view-based dimensionality reduction algorithms, such as PCA [2], LDA [2], LPP [3], MFA [10], DGPP [12], and the three latest multiview dimensionality reduction algorithms, including MVSE [18], MSNE [21], MSE [19], and SSMVE [22]. The nearest neighbor classifier with the Euclidean distance was adopted for classification. For a fair comparison, all the results reported here are based on the best tuned parameters of all the compared algorithms.

### 4.1. Data Sets and Experimental Settings

We conducted face recognition experiments on the widely used AR and CMU PIE face databases and facial expression recognition experiments on the well-known Japanese female facial expression (JAFFE) database.

**Figure 1:**
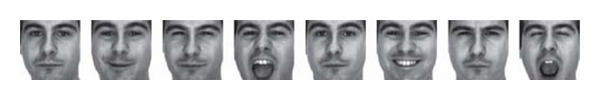
Sample face images from the AR database.

The AR database [24] contains over 4,000 color images corresponding to 126 people (70 men and 56 women), which include frontal view faces with different facial expressions, illumination conditions, and occlusions (sun glasses and scarf). Each person has 26 different images taken in two sessions (separated by two weeks). In our experiments, we used a subset of 800 face images from 100 persons (50 men and 50 women) with eight face images of different expressions and lighting conditions per person.  Figure 1 shows eight sample images of one individual from the subset of the AR database.

**Figure 2:**
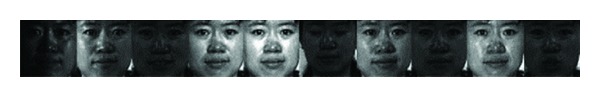
Sample face images from the CMU PIE database.

The CMU PIE database [25] comprises more than 40,000 facial images of 68 people with different poses, illumination conditions, and facial expressions. In this experiment, we selected a subset of the CMU PIE database which consists of 3060 frontal face images with varying expression and illumination from 68 persons with 45 images from each person.  Figure 2 shows some sample images of one individual from the subset of the CMU PIE database.

**Figure 3:**
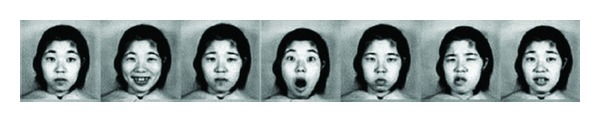
Sample facial images from the JAFFE database.

The Japanese female facial expression (JAFFE) database [26] contains 213 facial images of ten Japanese women. Each facial image shows one of seven expressions: neutral, happiness, sadness, surprise, anger, disgust, or fear.  Figure 3 shows some facial images from the JAFFE database. In this experiment, following the general setting scheme of facial expression recognition, we discard all the neutral facial images and only utilize the remainder 183 facial images which include six basic facial expressions.

For all the face images in the above three face databases, the facial part of each image was manually aligned, cropped, and resized into 32 × 32 according to the eye's positions. For each facial image, we extract the commonly used four kinds of low-level visual features to represent four different views. These four features include color histogram (CH) [27], scale-invariant feature transform (SIFT) [28], Gabor [29], and local binary pattern (LBP) [30]. For the CH feature extraction, we used 64 bins to encode a histogram feature for each facial image according to [27]. For the SIFT feature extraction, we densely sampled and calculated the SIFT descriptors of 16 × 16 patches over a grid with spacing of 8 pixels according to [28]. For the Gabor feature extraction, following [29], we adopted 40 Gabor kernel functions from five scales and eight orientations. For the LBP feature extraction, we followed the parameter settings in [30] and utilized 256 bins to encode a histogram feature for each facial image. For more details on these four feature descriptors, please refer to [27–30]. Because these four features are complementary to each other in representing facial images, we empirically set the tuning parameter *r* in MDGPP to be five.

In this experiment, each facial image set was partitioned into the nonoverlap training and testing sets. For each database, we randomly selected 50% data as the training set and use the remaining 50% data as the testing set. To reduce statistical variation for each random partition, we repeated these trials independently ten times and reported the average recognition results.

### 4.2. Compared Algorithms

We compared our proposed MDGPP algorithm with the following dimensionality reduction algorithms.

1. PCA [2]: PCA is an unsupervised dimensionality reduction algorithm.
2. LDA [2]: LDA is a supervised dimensionality reduction algorithm. We adopted a Tikhonov regularization term *μI* rather than PCA preprocessing to avoid the well-known small sample size (singularity) problem in LDA.
3. LPP [3]: LPP is an unsupervised manifold learning algorithm. There is a nearest neighbor number *k* to be tuned in LPP and it was empirically set to be five in our experiments. In addition, the Tikhonov regularization was also adopted to avoid the small sample size (singularity) problem in LPP.
4. MFA [10]: MFA is a supervised manifold learning algorithm. There are two parameters (i.e., *k*_1_ nearest neighbor number and *k*_2_ nearest neighbor number) to be tuned in MFA. We empirically set *k*_1_ = 5 and *k*_2_ = 20 in our experiments. Meanwhile, the Tikhonov regularization was also adopted to avoid the small sample size (singularity) problem in MFA.
5. DGPP [12]: DGPP is a supervised manifold learning algorithm. There is a tradeoff parameter *λ* to be tuned in DGPP and it was empirically set to be one in our experiments.
6. MVSE [18]: MSVE is an initially proposed multiview algorithm for dimensionality reduction.
7. MSNE [21]: MSNE is a probability-based unsupervised multiview algorithm. We followed the parameter setting in [21] and set the tradeoff coefficient *λ* to be five in our experiments.
8. MSE [19]: MSE is a supervised multiview algorithm. There are two parameters (i.e., the nearest neighbor number *k* and the tuning coefficient *r*) to be tuned in MSE. We empirically set *k* = 5 and *r* = 5 in our experiments.
9. SSMVE [22]: SSMVE is a sparse unsupervised multiview algorithm. We followed the parameter setting method in [22] and set the regularized parameter *λ* to be one in our experiments.

It is worth noting that since PCA, LDA, LPP, MFA, and DGPP are all single-view-based algorithms, these five algorithms adopt the conventional feature concatenation-based strategy to cope with the multiview data.

### 4.3. Experimental Results

**Figure 4:**
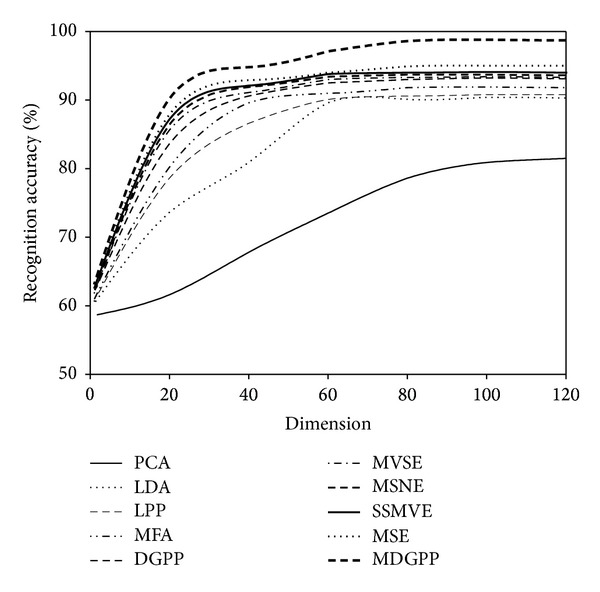
Recognition accuracy versus reduced dimension on the AR database.

**Figure 5:**
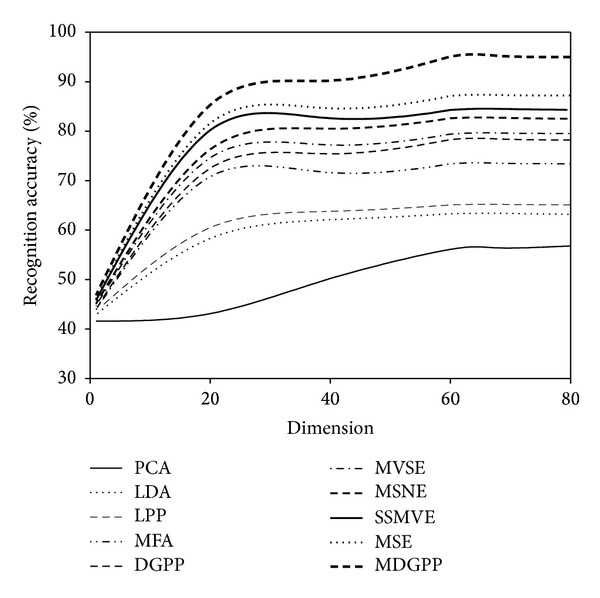
Recognition accuracy versus reduced dimension on the CMU PIE database.

**Figure 6:**
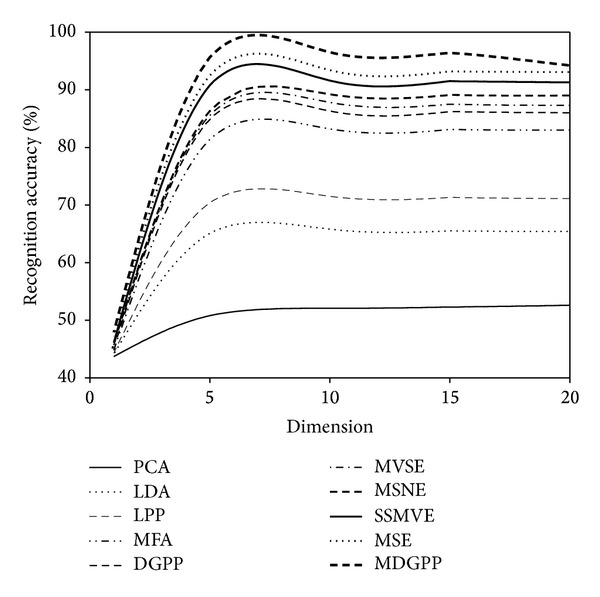
Recognition accuracy versus reduced dimension on the JAFFE database.

For each face image database, the recognition performance of different algorithms was evaluated on the testing data separately. The conventional nearest neighbor classifier with the Euclidean distance was applied to perform recognition in the subspace derived from different dimensionality reduction algorithms. Tables 1, 2, and 3 report the recognition accuracies and the corresponding optimal dimensions obtained on the AR, CMU PIE, and JAFFE databases, respectively. Figures 4, 5, and 6 illustrate the recognition accuracies versus the variation of reduced dimensions on the AR, CMU PIE, and JAFFE databases, respectively. According to the above experimental results, we can make the following observations.

1. As can be seen from Tables 1, 2, and 3 and Figures 4, 5, and 6, our proposed MDGPP algorithm consistently outperforms the conventional single-view-based algorithms (i.e., PCA, LDA, LPP, MFA, and DGPP) and the latest multiview algorithms (i.e., MVSE, MSNE, MSE, and SSMVE) in all the experiments, which implies that extracting a discriminative feature subspace by using both intraclass geometry and interclass discrimination and explicitly considering the complementary information of different facial features can achieve the best recognition performance.
2. The multiview learning algorithms (i.e., MVSE, MSNE, MSE, SSMVE, and MDGPP ) perform much better than single-view-based algorithms (i.e., PCA, LDA, LPP, MFA, and DGPP), which demonstrates that simple concatenation strategy cannot duly combine features from multiple views, and the recognition performance can be successfully improved by exploring the complementary characteristics of different views.
3. For the single-view-based algorithms, the manifold learning algorithms (i.e., LPP, MFA, and DGPP) perform much better than the conventional dimensionality reduction algorithms (i.e., PCA and LDA). This observation confirms that the local manifold structure information is crucial for image classification. Moreover, the supervised manifold learning algorithms (i.e., MFA and DGPP) perform much better than the unsupervised manifold learning algorithm LPP, which demonstrates that the utilization of discriminant information is useful to improve the image classification performance.
4. For the multiview learning algorithms, the supervised multiview algorithms (i.e., MSE and MDGPP) outperform the unsupervised multiview algorithms (i.e., MVSE, MSNE, and SSMVE) due to the utilization of the labeled facial images.
5. Although MVSE, MSNE, and SSMVE are all unsupervised multiview learning algorithms, SSMVE performs much better than MVSE and MSNE. The possible explanation is that the SSMVE algorithm adopts the sparse coding technique, which is naturally discriminative in determining the appropriate combination of different views.
6. Among the compared multiview learning algorithms, MVSE performs the worst. The reason is that MVSE performs a dimensionality reduction process on each view independently. Hence it cannot fully integrate the complementary information of different views to produce a good low-dimensional embedding.
7. MDGPP can improve the recognition performance of DGPP. The reason is that MDGPP can make use of multiple facial feature representations in a common learned subspace such that some complementary information can be explored for recognition task.

### 4.4. Convergence Analysis

**Figure 7:**
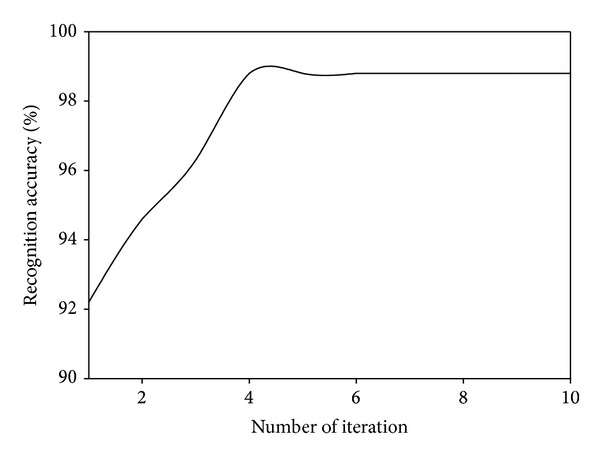
Recognition accuracy of MDGPP versus different numbers of iteration on the AR database.

**Figure 8:**
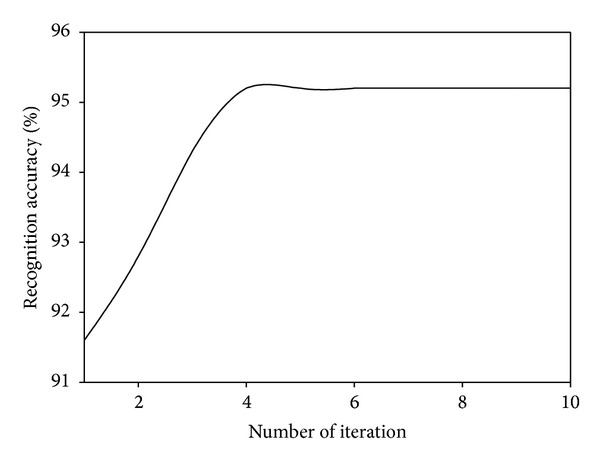
Recognition accuracy of MDGPP versus different numbers of iteration on the CMU PIE database.

**Figure 9:**
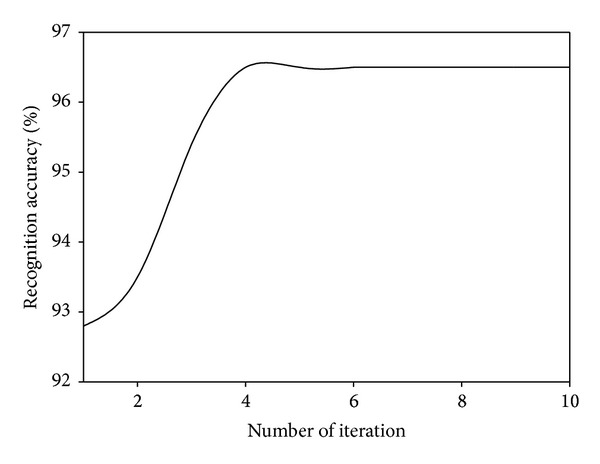
Recognition accuracy of MDGPP versus different numbers of iteration on the JAFFE database.

Since our proposed MDGPP is an iteration algorithm, we also evaluate its recognition performance with different numbers of iteration. Figures 7, 8, and 9 show the recognition accuracy of MDGPP versus different numbers of iteration on the AR, CMU PIE, and JAFFE databases, respectively. As can be seen from these figures, we can observe that our proposed MDGPP algorithm can converge to a local optimal optimum value in less than five iterations.

### 4.5. Parameter Analysis

**Figure 10:**
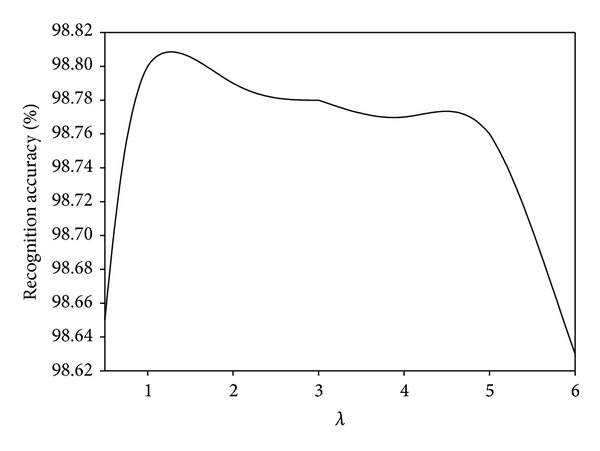
Recognition accuracy of MDGPP versus varying parameter *λ* on the AR database.

**Figure 11:**
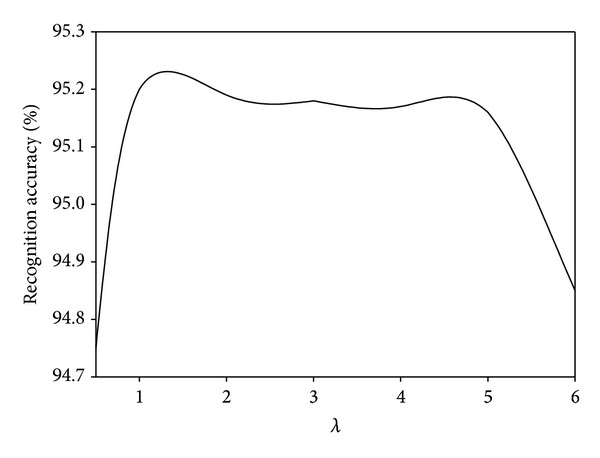
Recognition accuracy of MDGPP versus varying parameter *λ* on the CMU PIE database.

**Figure 12:**
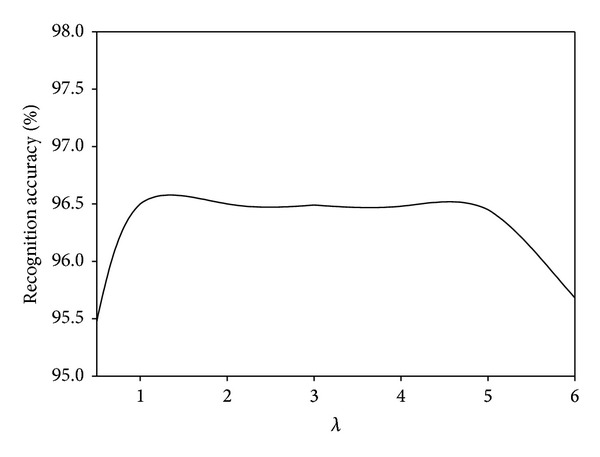
Recognition accuracy of MDGPP versus varying parameter *λ* on the JAFFE database.

**Figure 13:**
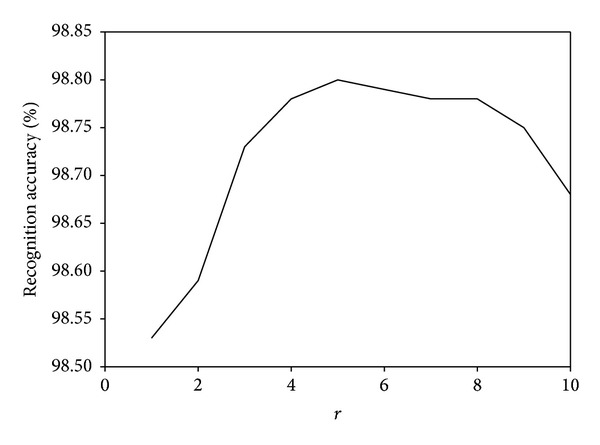
Recognition accuracy of MDGPP versus varying parameter *r* on the AR database.

**Figure 14:**
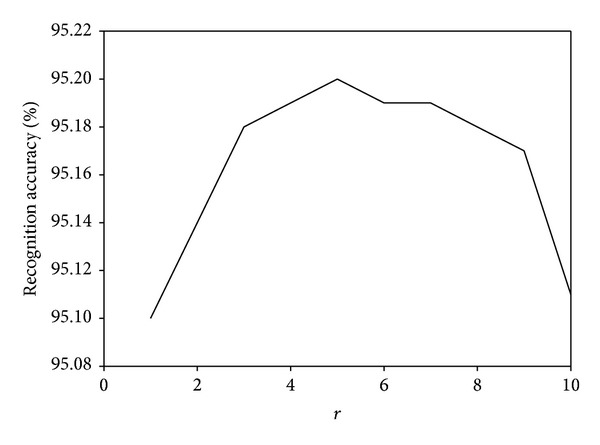
Recognition accuracy of MDGPP versus varying parameter *r* on the CMU PIE database.

**Figure 15:**
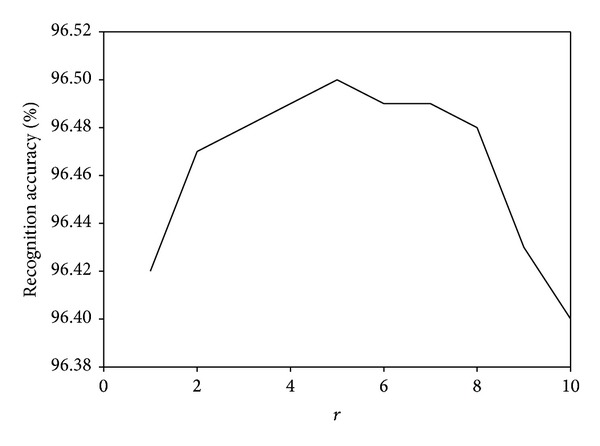
Recognition accuracy of MDGPP versus varying parameter *r* on the JAFFE database.

We investigate the parameter effects of our proposed MDGPP algorithm: tradeoff coefficient *λ* and tuning parameter *r*. Since each parameter can affect the recognition performance, we fix one parameter as used in the previous experiments and test the effect of the remaining one. Figures 10, 11, and 12 show the influence of the parameter *λ* in the MDGPP algorithm on the AR, CMU PIE, and JAFFE databases, respectively. Figures 13, 14, and 15 show the influence of the parameter *r* in the MDGPP algorithm on the AR, CMU PIE, and JAFFE databases, respectively. From Figure 10 to Figure 15, we can observe that MDGPP demonstrates a stable recognition performance over a large range of both *λ* and *r*. Therefore, we can conclude that the performance of MDGPP is not sensitive to the parameters *λ* and *r*.

## 5. Conclusion

In this paper, we have proposed a new multiview learning algorithm, called multiview discriminative geometry preserving projection (MDGPP) for feature extraction and classification by exploring the complementary property of different views. MDGPP can encode different features from different views in a physically meaningful subspace and learn a low-dimensional and sufficiently smooth embedding over all views simultaneously with an alternating-optimization-based iterative algorithm. Experimental results on three face image databases show that the proposed MDGPP algorithm outperforms other multiview and single view learning algorithms.

## Figures and Tables

**Table 1:** Comparisons of recognition accuracy on the AR database.

Algorithm	Accuracy	Dimensions
PCA	81.5%	120
LDA	90.4%	99
LPP	90.8%	100
MFA	91.9%	100
DGPP	93.2%	100
MVSE	93.4%	100
MSNE	93.7%	100
SSMVE	94.1%	100
MSE	95.0%	100
MDGPP	98.8%	100

**Table 2:** Comparisons of recognition accuracy on the CMU PIE database.

Algorithm	Accuracy	Dimensions
PCA	56.8%	80
LDA	63.4%	67
LPP	65.2%	68
MFA	73.5%	68
DGPP	78.4%	68
MVSE	79.6%	68
MSNE	82.7%	68
SSMVE	84.5%	68
MSE	87.3%	68
MDGPP	95.2%	68

**Table 3:** Comparisons of recognition accuracy on the JAFFE database.

Algorithm	Accuracy	Dimensions
PCA	52.6%	20
LDA	65.8%	9
LPP	71.5%	10
MFA	83.2%	10
DGPP	86.3%	10
MVSE	87.8%	10
MSNE	89.2%	10
SSMVE	91.6%	10
MSE	93.4%	10
MDGPP	96.5%	10
